# Hepatitis B virus pre-S2 deletion (nucleotide 1 to 54) in plasma predicts recurrence of hepatocellular carcinoma after curative surgical resection

**DOI:** 10.1371/journal.pone.0242748

**Published:** 2020-11-25

**Authors:** Chiao-Fang Teng, Tsai-Chung Li, Hsi-Yuan Huang, Wen-Ling Chan, Han-Chieh Wu, Woei-Cherng Shyu, Ih-Jen Su, Long-Bin Jeng

**Affiliations:** 1 Graduate Institute of Biomedical Sciences, China Medical University, Taichung, Taiwan; 2 Organ Transplantation Center, China Medical University Hospital, Taichung, Taiwan; 3 Research Center for Cancer Biology, China Medical University, Taichung, Taiwan; 4 Department of Public Health, College of Public Health, China Medical University, Taichung, Taiwan; 5 Department of Healthcare Administration, College of Medical and Health Science, Asia University, Taichung, Taiwan; 6 Department of Laboratory Medicine, China Medical University Hospital, Taichung, Taiwan; 7 Department of Bioinformatics and Medical Engineering, Asia University, Taichung, Taiwan; 8 Epigenome Research Center, China Medical University Hospital, Taichung, Taiwan; 9 National Institute of Infectious Diseases and Vaccinology, National Health Research Institutes, Zhunan, Taiwan; 10 Department of Occupational Therapy, Asia University, Taichung, Taiwan; 11 Department of Neurology, China Medical University Hospital, Taichung, Taiwan; 12 Translational Medicine Research Center, China Medical University Hospital, Taichung, Taiwan; 13 Department of Biotechnology, Southern Taiwan University of Science and Technology, Tainan, Taiwan; University of Cincinnati College of Medicine, UNITED STATES

## Abstract

Hepatocellular carcinoma (HCC) is a leading cause of cancer-related death worldwide. Despite curative surgical resection, high recurrence of HCC after surgery results in poor patient survival. To develop prognostic markers is therefore important for better prevention and therapy of recurrent HCC to improve patient outcomes. Deletion mutations over the pre-S1 and pre-S2 gene segments of hepatitis B virus (HBV) have been closely associated with recurrence of HCC after curative surgical resection. In this study, we applied a next-generation sequencing-based approach to further evaluate the association of pre-S deletion regions with HCC recurrence. We demonstrated that the pre-S2 deletion (nucleotide 1 to 54) was the most predominant deletion regions of pre-S gene in plasma of HBV-related HCC patients. Moreover, patients with the pre-S2 deletion (nucleotide 1 to 54) exhibited a significantly higher risk of HCC recurrence after curative surgical resection than those without. The pre-S2 deletion (nucleotide 1 to 54) in plasma represented a prognostic factor that independently predicted HCC recurrence with greater performance than other clinicopathological and viral factors. Our data suggest that detection of the pre-S2 deletion (nucleotide 1 to 54) in plasma may be a promising noninvasive strategy for identifying patients at high risk for HCC recurrence after curative surgical resection.

## Introduction

Among the most common and deadly human cancers worldwide, hepatocellular carcinoma (HCC) accounts for at least 700,000 deaths annually [1–3]. Although curative surgical resection of HCC is available [4–6], the 5-year recurrence rate of HCC after surgery remains up to 80%, resulting in poor patient survival [7–9]. Therefore, development of biomarkers for predicting HCC recurrence risk after surgical resection is important in allowing for early prevention and timely treatment of the recurrent HCC to ameliorate patient outcomes.

Chronic infection with hepatitis B virus (HBV) is intimately associated with the development of HCC, responsible for as high as 50% of HCC cases in the world [10–12]. In chronic HBV infection, ground glass hepatocytes (GGHs) in liver tissues represent preneoplastic lesions of HCC [13]. Two types of GGHs (designated type I and II) are identified to consistently express the pre-S1 and pre-S2 mutant proteins, respectively, which contain deletion mutations in the pre-S1 and pre-S2 gene segments of HBV large surface proteins [14, 15]. Both types of pre-S mutants have been well demonstrated to dysregulate various oncogenic signaling pathways, leading to tumorigenesis of hepatocytes and eventually HCC development [13, 16–18]. Chronic HBV-infected patients and HBV-related HCC patients who harbor pre-S mutants exhibit significantly higher incidences of HCC development [19–21] and recurrence after curative surgical resection [22–27], respectively. As a result, the presence of pre-S mutants is proposed as a valuable biomarker for the prognosis of HBV-related HCC.

Several approaches have been applied to qualitatively and semi-quantitatively detect pre-S mutants in blood and liver tissue specimens of HBV-related patients, including the approaches utilizing immunohistochemistry staining [15, 22], polymerase chain reaction (PCR) [19, 20, 28], and the Pre-S Gene Chip [21, 24]. Recently, we have developed a new approach based on next-generation sequencing (NGS) for quantitative detection of pre-S mutants from patient plasma with superior sensitivity, efficiency, and fidelity [29, 30]. In this study, we further evaluated the association between deletion regions of pre-S mutants and HCC recurrence in a cohort of 75 HBV-related HCC patients receiving curative surgical resection.

## Materials and methods

### Patient specimen collection

In this study, the HCC patients who had HBV infection and received curative surgical resection were included; the HCC patients who had infection with other types of hepatitis viruses and did not receive resection surgery were excluded. The plasma samples were collected from 75 HBV-related HCC patients who underwent surgery at the China Medical University Hospital (Taichung, Taiwan) from March 2004 to September 2016 under the approval of the China Medical University & Hospital Research Ethics Committee (protocol No. CMUH107-REC1-175). The written informed consent was obtained from all patients before surgery. The decoded clinicopathological data were acquired from the Human Biobank of the China Medical University Hospital. All research was conducted according to the relevant guidelines and regulations at the China Medical University Hospital.

### Detection of pre-S deletions by NGS

The NGS-based pre-S genotyping was carried out as described [29]. Briefly, patient plasma was used for the isolation of HBV DNA with the DNeasy Blood Kit (Qiagen, Valencia, CA, USA) according to the manufacturer’s instructions. The HBV DNA was served as the template for PCR-based amplification of the pre-S gene (comprising pre-S1 and pre-S2 gene segments). The PCR reaction was performed with the high-fidelity Platinum SuperFi DNA polymerase (Invitrogen, Carlsbad, CA, USA) together with specific primer pairs [first round: forward, 5’-GCGGGTCACCATATTCTTGGG-3’ (corresponding to nucleotide (nt) 2818 to 2837 in HBV genome sequence) and reverse, 5’-GAGTCTAGACTCTGCGGTAT-3’ (corresponding to nt 236 to 255 in HBV genome sequence); second round: forward, 5’-GCGGGTCACCATATTCTTGGG-3’ (corresponding to nt 2818 to 2837 in HBV genome sequence) and reverse, 5’-TAACACGAGCAGGGGTCCTA-3’ (corresponding to nt 180 to 199 in HBV genome sequence)] following the program [stage 1: 95°C for 1 minute; stage 2 (35 cycles): 95°C for 1 minute, 55°C for 1 minute, and 72°C for 1 minute; stage 3: 72°C for 7 minutes]. Next, the PCR products of pre-S gene (approximately ranging from 350 to 600 base pairs in size) were analyzed by NGS on the NextSeq 500 system (Illumina, San Diego, CA, USA) according to the manufacturer’s instructions in the Department of Laboratory Medicine at the China Medical University Hospital. All of the sequence reads were compared with the master sequences from the reference sets of HBV genotypes, which were available on the NCBI website (https://www.ncbi.nlm.nih.gov/projects/genotyping/view.cgi?db=2), by using BLAST. The deletion types, regions, and percentages of pre-S gene were determined by using our customized scripts. Finally, the pre-S deletion regions with the highest percentage in each type were identified for statistical analysis. S1 Table summarized the pre-S genotyping results of all patients.

### Statistical analysis

The univariate and multivariate analyses of pre-S deletions for overall (OS) and recurrence-free survival (RFS) were conducted by the Cox proportional-hazards regression model. The OS and RFS curves were analyzed by the Kaplan-Meier method and the log-rank test. The receiver operating characteristic (ROC) curves of pre-S deletions were established for discriminating patients with HCC recurrence from those without and the area under the ROC curves (AUCs) were determined and compared by the Hanley-McNeil test. All analyses were performed using SAS version 9.4 (SAS Institute Inc., Cary, NC, USA) [31]. A *P* value < 0.05 indicated a statistically significant difference.

## Results

### Clinicopathological data of 75 HBV-related HCC patients

As shown in Table 1, among all patients studied, 68 (91%) were men and 7 (9%) were women; 60 (80%) had HBV genotype B and 15 (20%) had genotype C; the median age was 53 years (range, 26 to 78); the median tumor size was 4.5 cm (range, 1.1 to 19.5). All patients received curative surgical resection, among whom 52 (69%) developed HCC recurrence and 16 (21%) died of disease after surgery; the median OS and RFS were 26.9 months (range, 6.8 to 161.1) and 11.2 months (range, 1.5 to 72.3), respectively (Table 1).

**Table 1:** Clinicopathological characteristics of 75 HBV-related HCC patients.

Characteristics	No. of Patients	Median (Range)
Age (years)	75	53 (26–78)
>50	48	60 (51–78)
≤50	27	43 (26–50)
Gender (men/women)	68/7	
Smoking (yes/no)	31/44	
Alcohol (yes/no)	29/46	
HBsAg (positive/negative/NA)	65/0/10	
HBeAg (positive/negative/NA)	9/62/4	
HBV genotype (B/C)	60/15	
HBV DNA (copies/mL) (20–1.7×10^8^/<20)^a^	74/1	2.1×10^4^ (21.5–1.5×10^8^)^c^
>1×10^4^	42	4.3×10^5^ (1.2×10^4^−1.5×10^8^)
≤1×10^4^	32	8.4×10^2^ (21.5–9.3×10^3^)
Albumin (g/dL)	75	3.7 (1.2–4.9)
>3.8	30	4.2 (3.9–4.9)
≤3.8	45	3.3 (1.2–3.8)
AST (U/L)	75	60 (14–1052)
>34	61	79 (35–1052)
≤34	14	27 (14–34)
ALT (U/L)	75	55 (13–1338)
>40	50	96.5 (41–1338)
≤40	25	31 (13–40)
AFP (ng/mL) (≤54000/>54000)^b^	71/4	26.7 (1.8–36600.0)^d^
>400	28	1920 (461.7–36600.0)
≤400	47	13.8 (1.8–271.0)
Tumor size (cm)	75	4.5 (1.1–19.5)
>5	37	10.0 (5.5–19.5)
≤5	38	2.4 (1.1–4.5)
Tumor encapsulation (yes/no/NA)	42/20/13	
Lymph node involvement (yes/no)	Aug-67	
Portal vein thrombosis (yes/no)	May-70	
Vascular invasion (yes/no)	27/48	
Distant metastasis (yes/no)	Aug-67	
Steatosis grade (0/1/2/3/NA)	14/10/1/0/50	
Metavir inflammation score (0/1/2/3/NA)	4/35/5/0/31	
Ishak fibrosis score (0/1/2/3/4/5/6/NA)	5/13/12/8/3/4/11/19	
Child-Pugh cirrhosis score (A/B/C)	57/16/2	
CLIP score (0/1/2/3/4/5/6)	33/23/10/8/1/0/0	
BCLC stage (A/B/C/D)	38/29/7/1	
AJCC TNM stage (I/II/IIIA/IIIB/IIIC/IVA/IVB)	40/20/7/5/3/0/0	
Antiviral therapy after surgery (yes/no)	40/35	
HCC recurrence after surgery (month) (yes/no)	52/23	11.2 (1.5–72.3)^e^
Survival after surgery (month) (dead/alive)	16/59	26.9 (6.8–161.1)^f^

^a^HBV DNA was measured with a detection range of 20 to 1.7×10^8^ copies/mL.

^b^AFP was measured with the highest detection limit of 54000 ng/mL.

^c,d^Only data within the detection range were analyzed.

^e^Shown was the time to recurrence after surgery.

^f^Shown was survival time in patients who died after surgery.

Abbreviations: HBV, hepatitis B virus; HCC, hepatocellular carcinoma; HBeAg, hepatitis B e antigen; NA, not available; AST, aspartate aminotransferase; ALT, alanine aminotransferase; AFP, alpha-fetoprotein; CLIP, Cancer of the Liver Italian Program; BCLC, Barcelona Clinic Liver Cancer; AJCC, American Joint Committee on Cancer; TNM, tumor-node-metastasis.

### Profiles of deletion regions of pre-S gene in patient plasma

**Fig 1:**
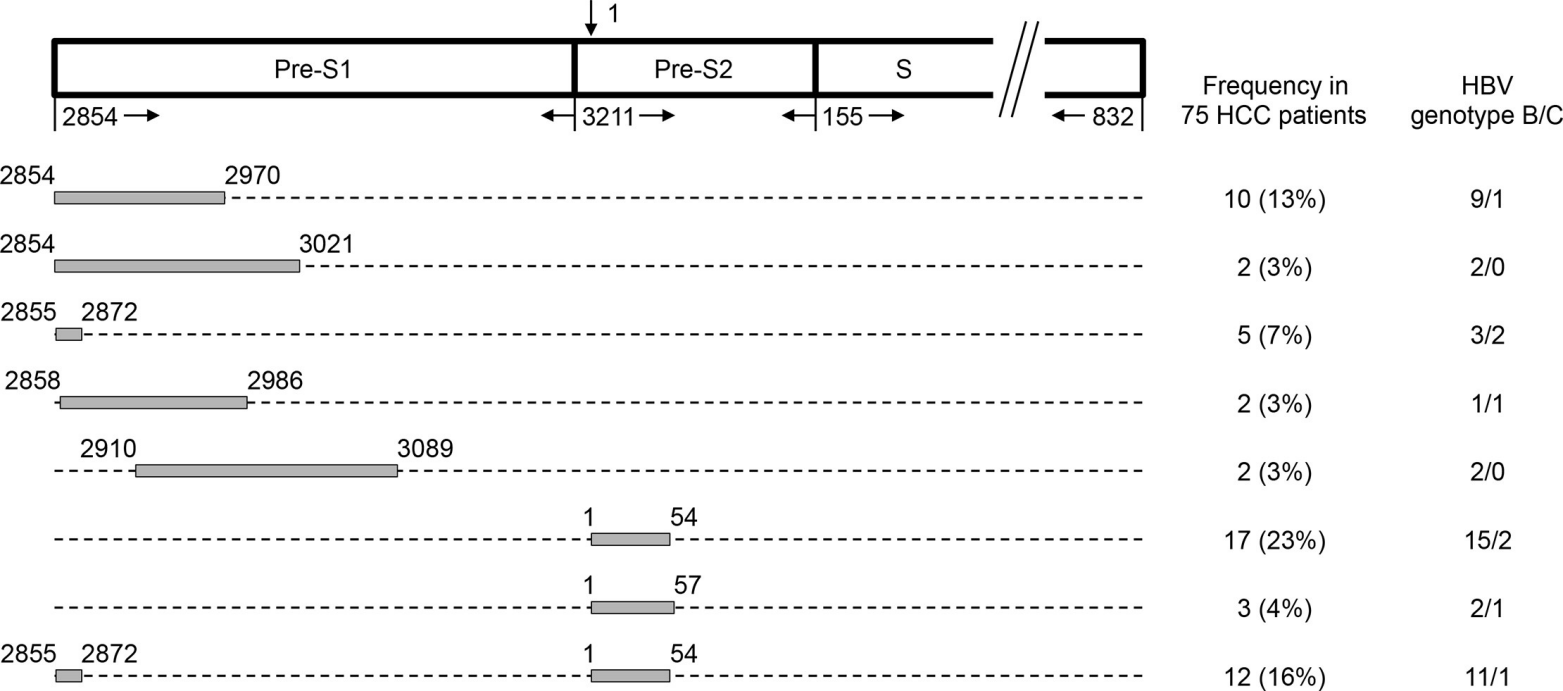
Schematic summary of the pre-S deletion regions in plasma of 75 HBV-related HCC patients. The HBV surface gene is composed of the pre-S1, pre-S2, and S gene segments. The arrow above the diagram indicates the start site (nt 1) of the circular HBV genome that goes clockwise and ends at nt 3221 (not shown). The nt numbers below the diagram indicate the positions of three gene segments in the HBV genome. The grey boxes represent the deletion regions detected in the pre-S1 and pre-S2 gene segments. Here only the deletion regions found in at least 2 of 75 patients were shown and designated in order from the top to bottom as follows: the pre-S1 deletion (nt 2854 to 2970), the pre-S1 deletion (nt 2854 to 3021), the pre-S1 deletion (nt 2855 to 2872), the pre-S1 deletion (nt 2858 to 2986), the pre-S1 deletion (nt 2910 to 3089), the pre-S2 deletion (nt 1 to 54), the pre-S2 deletion (nt 1 to 57), and the pre-S1+pre-S2 deletion (nt 2855 to 2872, 1 to 54). The frequency of these pre-S deletion regions in 75 HCC patients as well as the number of patients with HBV genotype B or C for each type of pre-S deletion region were shown on the right half of the figure. Abbreviations: HCC, hepatocellular carcinoma.

As shown in Fig 1 and Table 2, among all patients analyzed, 46 (61%) had pre-S deletions, among whom 10 (13%) had the pre-S1 deletion (nt 2854 to 2970), 2 (3%) had the pre-S1 deletion (nt 2854 to 3021), 5 (7%) had the pre-S1 deletion (nt 2855 to 2872), 2 (3%) had the pre-S1 deletion (nt 2858 to 2986), 2 (3%) had the pre-S1 deletion (nt 2910 to 3089), 17 (23%) the had pre-S2 deletion (nt 1 to 54), 3 (4%) had the pre-S2 deletion (nt 1 to 57), and 12 (16%) had the pre-S1+pre-S2 deletion (nt 2855 to 2872, 1 to 54). Several pre-S deletion regions were found in only 1 patient (S1 Table).

**Table 2:** Summary of pre-S deletion regions in 75 HBV-related HCC patients.

Summary of Pre-S Deletion Regions^a^	No. of Patients
Total patients (n) (%)	75 (100)
Patients without pre-S del (n) (%)	29 (39)
Patients with pre-S del (n) (%)	46 (83)
Patients with pre-S1 del (nt 2854–2970) (n) (%)	10 (13)
Patients with pre-S1 del (nt 2854–3021) (n) (%)	2 (3)
Patients with pre-S1 del (nt 2855–2872) (n) (%)	5 (7)
Patients with pre-S1 del (nt 2858–2986) (n) (%)	2 (3)
Patients with pre-S1 del (nt 2910–3089) (n) (%)	2 (3)
Patients with pre-S2 del (nt 1–54) (n) (%)	17 (23)
Patients with pre-S2 del (nt 1–57) (n) (%)	3 (4)
Patients with pre-S1+pre-S2 del (nt 2855–2872, 1–54) (n) (%)	12 (16)

^a^Only the pre-S deletion regions found in at least 2 of 75 patients were shown.

Abbreviations: n, number; del, deletion; nt, nucleotide.

### Patients with the pre-S2 deletion (nt 1 to 54) in plasma as a population at high risk for HCC recurrence after curative surgical resection

**Fig 2:**
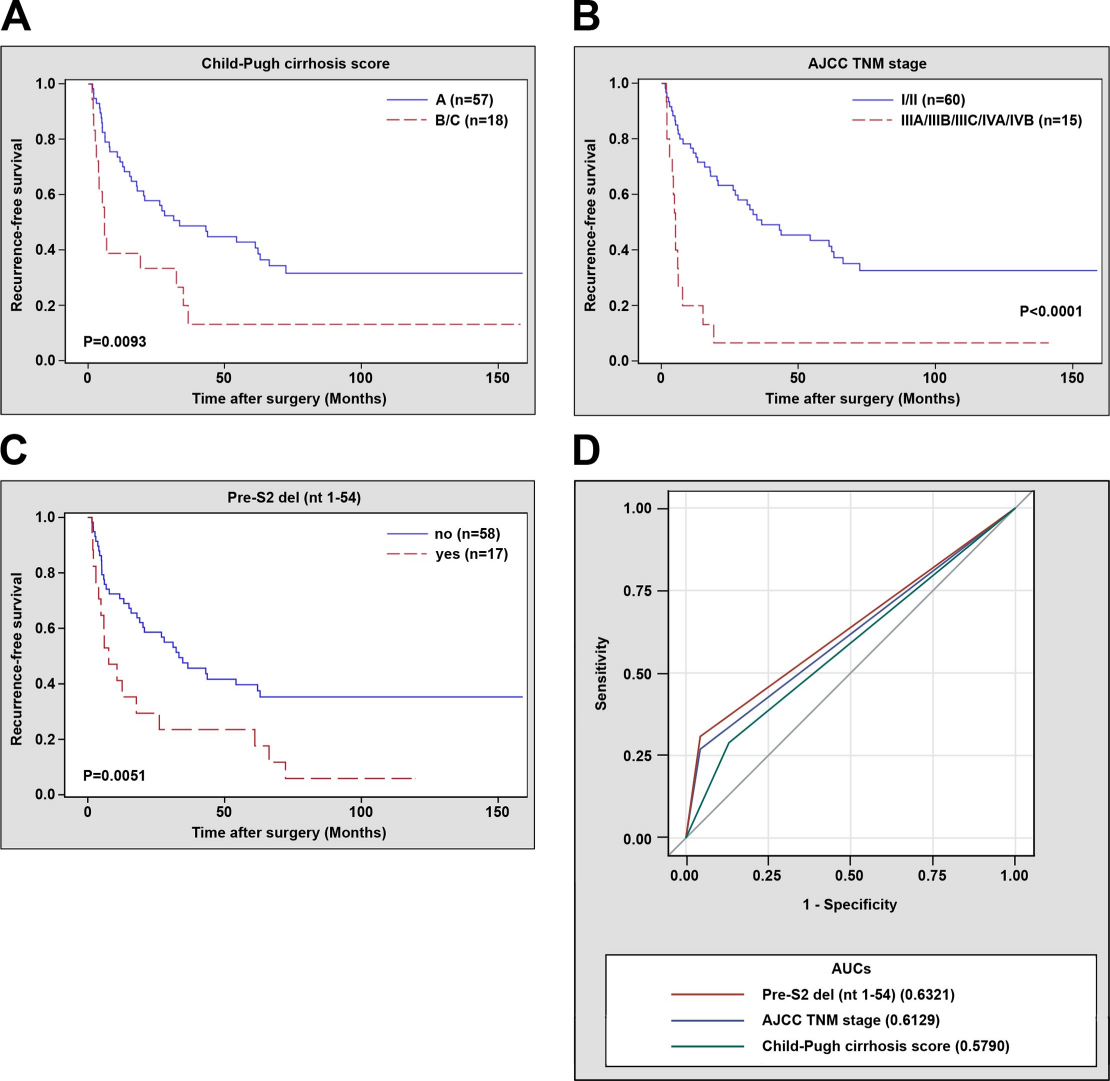
RFS and ROC curves in 75 HBV-related HCC patients receiving curative surgical resection. RFS curves in patients with Child-Pugh cirrhosis score B/C versus A (A), in patients with AJCC TNM stage IIIA/IIIB/IIIC/IVA/IVB versus I/II (B), and in patients with (yes) versus without (no) the pre-S2 deletion (nt 1–54) (C). *P* values and numbers (n) of patients were indicated in the plots. A *P* value < 0.05 indicated a statistically significant difference. (D) ROC curves in discriminating patients with HCC recurrence from those without. AUCs for the prognostic factors, including the pre-S2 deletion (nt 1–54) (red line), Child-Pugh cirrhosis score (green line), and AJCC TNM stage (blue line) were indicated in the plot. Abbreviations: n, number; AJCC, American Joint Committee on Cancer; TNM, tumor-node-metastasis; del, deletion; nt, nucleotide; AUCs, area under the receiver operating characteristic curves.

The deletion regions of pre-S gene whose percentages in patient plasma ranked among the top in each type were selected for clinicopathological analysis, including the pre-S1 deletion (nt 2854 to 2970), the pre-S1 deletion (nt 2855 to 2872), the pre-S2 deletion (nt 1 to 54), and the pre-S1+pre-S2 deletion (nt 2855 to 2872, 1 to 54). Among these deletion regions, only the pre-S2 deletion (nt 1 to 54) had a significantly positive correlation with HCC recurrence (*P* value = 0.0080) (Table 3). Moreover, the pre-S2 deletion (nt 1 to 54), along with the Child-Pugh cirrhosis score and AJCC TNM stage, were significantly and independently associated with poor RFS in patients (pre-S2 deletion (nt 1 to 54), hazard ratio (HR) = 2.392, 95% confidence interval (CI) 1.297 to 4.410, *P* value = 0.0052; Child-Pugh cirrhosis score, HR = 2.065, 95% CI 1.086 to 3.927, *P* value = 0.0271; AJCC TNM stage, HR = 3.411, 95% CI 1.710 to 6.804, *P* value = 0.0005) (Table 4). Patients with the pre-S2 deletion (nt 1 to 54), Child-Pugh cirrhosis score (B/C), or AJCC TNM stage (IIIA/IIIB/IIIC/IVA/IVB) had a significantly shorter median RFS than those without (pre-S2 deletion (nt 1 to 54), 7.7 vs. 31.7 months, *P* value = 0.0283; Child-Pugh cirrhosis score, 5.1 vs. 15.1 months, *P* value = 0.0093; AJCC TNM stage, 5.0 vs. 17.9 months, *P* value<0.0001) (Fig 2A to 2C). However, there was no significant association between pre-S deletion regions and OS in patients (S2 and S3 Tables). Furthermore, the prognostic performance of these factors in predicting HCC recurrence was evaluated, showing that the pre-S2 deletion (nt 1 to 54) had the highest AUC (0.6321, 95% CI 0.5558 to 0.7084) followed by the AJCC TNM stage (0.6129, 95% CI 0.5386 to 0.6872) and the Child-Pugh cirrhosis score (0.5790, 95% CI 0.4851 to 0.6729) (Fig 2D).

**Table 3:** Clinicopathological correlation of pre-S deletion regions with HCC recurrence after surgery in 75 HBV-related HCC patients.

Characteristics^a^	Yes (No. of Patients (%))	No (No. of Patients (%))	P value^b^
Pre-S1 del (nt 2854–2970)	52 (100)	23 (100)	
yes	7 (13)	3 (13)	0.2858
no	45 (87)	20 (87)	
Pre-S1 del (nt 2855–2872)	52 (100)	23 (100)	
yes	4 (8)	1 (4)	0.3608
no	48 (92)	22 (96)	
Pre-S2 del (nt 1–54)	52 (100)	23 (100)	
yes	16 (31)	1 (4)	0.0080**
no	36 (69)	22 (96)	
Pre-S1+pre-S2 del (nt 2855–2872, 1–54)	52 (100)	23 (100)	
yes	11 (21)	1 (4)	0.0532
no	41 (79)	22 (96)	

^a^Only patients with available data were analyzed.

^b^P value was determined by the chi-square test.

**, P value<0.01.

Abbreviations: HCC, hepatocellular carcinoma; HBV, hepatitis B virus; del, deletion; nt, nucleotide.

**Table 4:** Univariate and multivariate analyses of pre-S deletion regions for recurrence-free survival in 75 HBV-related HCC patients.

Characteristics	Univariate Analysis			Multivariate Analysis		
	HR	95% CI	P value	HR	95% CI	P value
Age (years) (>50 vs. ≤50)	0.951	0.532–1.700	0.8666			
Gender (men vs. women)	1.043	0.414–2.627	0.9284			
Smoking (yes vs. no)	0.886	0.503–1.560	0.6750			
Alcohol (yes vs. no)	0.884	0.494–1.580	0.6773			
HBsAg (positive vs. negative)^a^						
HBeAg (positive vs. negative)^b^	1.234	0.523–2.910	0.6307			
HBV genotype (B vs. C)	0.583	0.304–1.117	0.1040			
HBV DNA (copies/mL) (>1×10^4^ vs. ≤1×10^4^)^c^	1.645	0.934–2.895	0.0846			
Albumin (g/dL) (>3.8 vs. ≤3.8)	0.551	0.288–1.092	0.0585			
AST (U/L) (>34 vs. ≤34)	0.865	0.444–1.684	0.6691			
ALT (U/L) (>40 vs. ≤40)	0.797	0.456–1.394	0.4267			
AFP (ng/mL) (>400 vs. ≤400)	1.305	0.745–2.285	0.3524			
Tumor size (cm) (>5 vs. ≤5)	1.490	0.863–2.572	0.1525			
Tumor encapsulation (yes vs. no)^d^	0.901	0.474–1.713	0.7508			
Lymph node involvement (yes vs. no)	0.333	0.104–1.071	0.0652			
Portal vein thrombosis (yes vs. no)	1.668	0.600–4.633	0.3264			
Vascular invasion (yes vs. no)	1.677	0.962–2.924	0.0681			
Distant metastasis (yes vs. no)	2.259	0.999–5.101	0.0502			
Steatosis grade (2/3 vs. 0/1)^e^	3.473	0.418–28.879	0.2493			
Metavir inflammation score (2/3 vs. 0/1)^f^	0.731	0.256–2.088	0.5583			
Ishak fibrosis score (4/5/6 vs. 0/1/2/3)^g^	1.261	0.670–2.373	0.4714			
Child-Pugh cirrhosis score (B/C vs. A)	2.189	1.195–4.013	0.0112*	2.065	1.086–3.927	0.0271*
CLIP score (4/5/6 vs. 0/1/2/3)	2.426	0.328–17.911	0.3850			
BCLC stage (C/D vs. A/B)	1.927	0.867–4.284	0.1077			
AJCC TNM stage (IIIA/IIIB/IIIC/IVA/IVB vs. I/II)	4.048	2.123–7.719	<0.0001***	3.411	1.710–6.804	0.0005***
Antiviral therapy after surgery (yes vs. no)	1.176	0.674–2.051	0.5684			
Pre-S1 del (nt 2854–2970) (yes vs. no)	1.166	0.525–2.593	0.7059			
Pre-S1 del (nt 2855–2872) (yes vs. no)	1.293	0.465–3.598	0.6221			
Pre-S2 del (nt 1–54) (yes vs. no)	2.279	1.260–4.122	0.0065**	2.392	1.297–4.410	0.0052**
Pre-S1+pre-S2 del (nt 2855–2872, 1–54) (yes vs. no)	1.614	0.827–3.151	0.1604			

^a^There were no patients negative for HBsAg for analysis.

^b^Only 71 patients with available data were analyzed.

^c^Only 74 patients with available data were analyzed.

^d^Only 62 patients with available data were analyzed.

^e^Only 25 patients with available data were analyzed.

^f^Only 44 patients with available data were analyzed.

^g^Only 56 patients with available data were analyzed.

*, P value<0.05

**, P value<0.01

***, P value<0.001.

Abbreviations: HR, hazard ratio; CI, confidence interval; del, deletion; vs., versus.

## Discussion

Although curative surgical resection is available for treating HCC patients, high recurrence rate of HCC after surgery is still a big threat, causing poor patient outcomes [32–34]. Patients carrying HBV pre-S mutants, which contain deletions over the pre-S1 and pre-S2 regions, have been demonstrated to be at high risk for HCC recurrence after curative surgical resection [22–25]. In this study, we further evaluated the association of pre-S deletion regions in plasma with HCC recurrence and identified the pre-S2 deletion (nt 1 to 54) as an independent prognostic biomarker for HCC recurrence with greater performance than other clinicopathological and viral factors.

Several reports have examined the deletion incidences and patterns of HBV pre-S gene in patients with different stages of chronic HBV infection-related liver diseases, including chronic hepatitis, liver cirrhosis, and HCC. The incidences of overall pre-S deletions (including the pre-S1 and pre-S2 deletions) are gradually increased from chronic hepatitis patients to liver cirrhosis patients and eventually reach a peak in HCC patients [13, 21, 28, 35–37]. The pre-S1 deletion is most frequently detected in liver cirrhosis patients, while the pre-S2 deletion is most frequently detected in HCC patients [35–37]. The distributions of the pre-S1 deletion regions nearly cover the entire pre-S1 gene segment with the highest prevalence in the former region, but the distributions of the pre-S2 deletion regions predominantly fall in the former region of pre-S2 gene segment [28, 35–37]. Moreover, patients with pre-S deletions show a significantly higher risk of developing liver cirrhosis and HCC than those without [19–21]. Consistent with these findings, in this study the pre-S genotyping results from plasma of HBV-related HCC patients displayed similar distribution patterns of pre-S deletion regions, among which the pre-S2 deletion (nt 1 to 54) had the highest incidence. Furthermore, we provided evidence supporting that patients with the pre-S2 deletion (nt 1 to 54) represented a population at high risk of HCC recurrence after curative surgical resection. Considering that the pre-S2 region (nt 1 to 54) coincides with the B- and T-cell epitopes of HBV large surface proteins [28, 38–40], the pre-S2 mutant proteins harboring the pre-S2 deletion (nt 1 to 54) may emerge as an immune escape mutant that may possibly explain their high incidence in HCC and high association with HCC recurrence after curative surgical resection.

The clinical correlation between HBV pre-S deletions and HCC recurrence after curative surgical resection has been well documented [41]. Both the presence and higher percentage of pre-S deletion mutations in liver tissues and serum/plasma of HCC patients have been independently associated with a higher risk of HCC recurrence after resection surgery [22–27]. However, before this study, the association of specific pre-S deletion regions with HCC recurrence remains poorly defined. In this study, we for the first time identified that the presence of the pre-S2 deletion (nt 1 to 54) in plasma of HCC patients was an independent biomarker for prediction of HCC recurrence after curative surgical resection. Our finding may potentially support the development of approaches toward focusing on detection of specific pre-S deletion regions, thus facilitating the clinical application of pre-S deletions as biomarkers in prediction of HCC recurrence. Although a previous report by Jia et al has shown that deletions in the pre-S gene display consistent patterns and incidences between the matched serum and liver tissues in HBV-related HCC patients [37], whether the pre-S2 deletion (nt 1 to 54) may be also detected in liver tissues of HCC patients to predict the recurrence of HCC after resection surgery still needs to be investigated. In addition, although the clinicopathological characteristics of the cohort of 75 HCC patients analyzed in this study coincided with the representative features of a large population of HCC patients in Taiwan [42], a large cohort of patients from different clinical centers are needed to further validate this finding in clinical practice.

## Conclusions

Our results demonstrated that patients with the HBV pre-S2 deletion (nt 1–54) in plasma had a higher risk of HCC recurrence than those without after curative surgical resection. Detection of the pre-S2 deletion (nt 1–54) in plasma may be a promising noninvasive strategy for identifying patients at high risk of postoperative HCC recurrence, allowing them for early preventive and timely therapeutic managements for better survival.

## Supporting information

S1 Table List of the pre-S genotyping results by NGS-based analysis in 75 HBV-related HCC patients. (DOCX)

S2 Table Clinicopathological correlation of pre-S deletion regions with survival after surgery in 75 HBV-related HCC patients. (DOCX)

S3 Table Univariate and multivariate analyses of pre-S deletion regions for overall survival in 75 HBV-related HCC patients. (DOCX)
