# Supplementary material for: Hepatitis B virus pre-S2 deletion (nucleotide 1 to 54) in plasma predicts recurrence of hepatocellular carcinoma after curative surgical resection
Source: PLoS One. 2020 Nov 25;15(11):e0242748. doi: 10.1371/journal.pone.0242748 (PMC7688176; doi:10.1371/journal.pone.0242748)
Supplement: S1 Table — (DOCX) [file pone.0242748.s001.docx]

**S1 Table.** **List of the pre-S genotyping results by NGS-based analysis in 75 HBV-related HCC patients**

| Patient No. | Pre-S Deletion Type (%)^a^ | Pre-S Deletion Region (%)^b^ |
| --- | --- | --- |
| 1 | 1. **pre-S1 del (92.118)**^c^  **2. wild-type (7.278)**  3. pre-S2 del (0.372)  4. pre-S1+pre-S2 del (0.231) | 1. pre-S1 del (nt 2855-2872) (90.102)  2. wild-type (7.278)  3. pre-S2 del (nt 1-28) (0.180)  4. pre-S1+pre-S2 del (nt 2855-2872, 1-111) (0.027) |
| 2 | 1. **pre-S1 del (75.241)**  2. **wild-type (22.338)**  3. pre-S2 del (1.891)  4. pre-S1+pre-S2 del (0.530) | 1. pre-S1 del (nt 2910-3089) (48.977)  2. wild-type (22.338)  3. pre-S2 del (nt 1-54) (1.579)  4. pre-S1+pre-S2 del (nt 2910-3089, 1-57) (0.277) |
| 3 | 1. **pre-S1 del (76.228)**  2. **wild-type (12.583)**  3. **pre-S2 del (10.622)**  4. pre-S1+pre-S2 del (0.567) | 1. pre-S1 del (nt 3110-3127) (24.869)  2. wild-type (12.583)  3. pre-S2 del (nt 1-57) (10.000)  4. pre-S1+pre-S2 del (nt 2855-2872, 1-57) (0.490) |
| 4 | 1. **wild-type (71.590)**  2. **pre-S1 del (28.270)**  3. pre-S2 del (0.129)  4. pre-S1+pre-S2 del (0.011) | 1. wild-type (71.590)  2. pre-S1 del (nt 2854-3147) (23.961)  3. pre-S2 del (nt 3211-3216) (0.048)  4. pre-S1+pre-S2 del (nt 2954-3097, 1-54) (0.002) |
| 5 | 1. **wild-type (58.461)**  2. **pre-S2 del (37.194)**  3. pre-S1 del (2.939)  4. pre-S1+pre-S2 del (1.407) | 1. wild-type (58.461)  2. pre-S2 del (nt 24-50) (33.801)  3. pre-S1 del (nt 2880-3146) (0.646)  4. pre-S1+pre-S2 del (nt 2880-3146, 24-50) (0.355) |
| 6 | 1. **wild-type (96.592)**  2. pre-S1 del (2.906)  3. pre-S2 del (0.469)  4. pre-S1+pre-S2 del (0.033) | 1. wild-type (96.592)  2. pre-S1 del (nt 3026-3205) (0.604)  3. pre-S2 del (nt 1-12) (0.233)  4. pre-S1+pre-S2 del (nt 2854-2979, 6-134) (0.008) |

**S1 Table. List of the pre-S genotyping results by TA cloning- and NGS-based analyses in 75 HBV-related HCC patients (continued)**

| Patient No. | NGS Result (Pre-S Del Type (%))^a^ | NGS Result (Pre-S Del Region (%))^b^ |
| --- | --- | --- |
| 7 | 1. **wild-type (99.000)**  2. pre-S1 del (0.927)  3. pre-S2 del (0.062)  4. pre-S1+pre-S2 del (0.012) | 1. wild-type (99.000)  2. pre-S1 del (nt 3106-3129) (0.294)  3. pre-S2 del (nt 1-54) (0.031)  4. pre-S1+pre-S2 del (nt 2855-2972, 1-54) (0.008) |
| 8 | 1. **wild-type (93.746)**  2. **pre-S1 del (5.846)**  3. pre-S2 del (0.389)  4. pre-S1+pre-S2 del (0.018) | 1. wild-type (93.746)  2. pre-S1 del (nt 3026-3205) (2.417)  3. pre-S2 del (nt 3211-3213) (0.135)  4. pre-S1+pre-S2 del (nt 2856-2873, 1-21) (0.004) |
| 9 | 1. **pre-S1+pre-S2 del (46.237)**  2. **pre-S2 del (26.927)**  3. **pre-S1 del (14.368)**  4. **wild-type (12.467)** | 1. pre-S1+pre-S2 del (nt 2956-3126, 1-9) (24.002)  2. pre-S2 del (nt 1-54) (23.226)  3. pre-S1 del (nt 2944-3075) (5.140)  4. wild-type (12.467) |
| 10 | 1. **wild-type (97.851)**  2. pre-S1 del (2.037)  3. pre-S2 del (0.108)  4. pre-S1+pre-S2 del (0.005) | 1. wild-type (97.851)  2. pre-S1 del (nt 3026-3205) (1.109)  3. pre-S2 del (nt 3211-3213) (0.049)  4. pre-S1+pre-S2 del (nt 2855-2872, 1-54) (0.003) |
| 11 | 1. **wild-type (98.964)**  2. pre-S1 del (0.943)  3. pre-S2 del (0.084)  4. pre-S1+pre-S2 del (0.009) | 1. wild-type (98.964)  2. pre-S1 del (nt 2854-2970) (0.125)  3. pre-S2 del (nt 1-9) (0.028)  4. pre-S1+pre-S2 del (nt 2954-3097, 1-13) (0.009) |
| 12 | 1. **wild-type (97.781)**  2. pre-S1 del (2.099)  3. pre-S2 del (0.112)  4. pre-S1+pre-S2 del (0.008) | 1. wild-type (97.781)  2. pre-S1 del (nt 3026-3205) (1.099)  3. pre-S2 del (nt 3211-3216) (0.055)  4. pre-S1+pre-S2 del (nt 3026-3205, 43-138) (0.002) |

**S1 Table. List of the pre-S genotyping results by TA cloning- and NGS-based analyses in 75 HBV-related HCC patients (continued)**

| Patient No. | NGS Result (Pre-S Del Type (%))^a^ | NGS Result (Pre-S Del Region (%))^b^ |
| --- | --- | --- |
| 13 | 1. **wild-type (92.291)**  2. pre-S1 del (4.092)  3. pre-S2 del (3.038)  4. pre-S1+pre-S2 del (0.578) | 1. wild-type (92.291)  2. pre-S1 del (nt 2854-2970) (0.650)  3. pre-S2 del (nt 1-54) (2.641)  4. pre-S1+pre-S2 del (nt 2855-2872, 1-54) (0.435) |
| 14 | 1. **wild-type (69.001)**  2. **pre-S1 del (20.530)**  3**. pre-S2 del (9.463)**  4. pre-S1+pre-S2 del (1.006) | 1. wild-type (69.001)  2. pre-S1 del (nt 3110-3127) (4.779)  3. pre-S2 del (nt 1-54) (8.226)  4. pre-S1+pre-S2 del (nt 2855-2872, 1-18, 26-59) (0.259) |
| 15 | 1. **wild-type (50.938)**  2. **pre-S1 del (19.760)**  3. **pre-S1+pre-S2 del (15.021)**  4. **pre-S2 del (14.280)** | 1. wild-type (50.938)  2. pre-S1 del (nt 2854-2970) (5.470)  3. pre-S1+pre-S2 del (nt 2855-2872, 1-54) (12.421)  4. pre-S2 del (nt 1-54) (13.606) |
| 16 | 1. **wild-type (75.230)**  2. **pre-S1+pre-S2 del (13.878)**  3. **pre-S1 del (10.730)**  4. pre-S2 del (0.162) | 1. wild-type (75.230)  2. pre-S1+pre-S2 del (nt 2860-2880, 2954-3097, 1-15) (11.572)  3. pre-S1 del (nt 2860-2880, 2954-3097) (9.550)  4. pre-S2 del (nt 1-15) (0.138) |
| 17 | 1. **wild-type (98.571)**  2. pre-S1 del (1.173)  3. pre-S2 del (0.178)  4. pre-S1+pre-S2 del (0.078) | 1. wild-type (98.571)  2. pre-S1 del (nt 3106-3129) (0.242)  3. pre-S2 del (nt 2-149) (0.043)  4. pre-S1+pre-S2 del (nt 2854-2985, 27-149) (0.014) |
| 18 | 1. **wild-type (96.915)**  2. pre-S1 del (2.701)  3. pre-S2 del (0.364)  4. pre-S1+pre-S2 del (0.020) | 1. wild-type (96.915)  2. pre-S1 del (nt 3026-3205) (1.755)  3. pre-S2 del (nt 25-54) (0.148)  4. pre-S1+pre-S2 del (nt 2854-2988, 45-149) (0.004) |

**S1 Table. List of the pre-S genotyping results by TA cloning- and NGS-based analyses in 75 HBV-related HCC patients (continued)**

| Patient No. | NGS Result (Pre-S Del Type (%))^a^ | NGS Result (Pre-S Del Region (%))^b^ |
| --- | --- | --- |
| 19 | 1. **wild-type (98.069)**  2. pre-S1 del (1.263)  3. pre-S2 del (0.609)  4. pre-S1+pre-S2 del (0.059) | 1. wild-type (98.069)  2. pre-S1 del (nt 3103-3126) (0.253)  3. pre-S2 del (nt 1-15) (0.535)  4. pre-S1+pre-S2 del (nt 2854-2996, 44-144) (0.015) |
| 20 | 1. **wild-type (69.571)**  2. **pre-S2 del (18.590)**  **3. pre-S1+pre-S2 del (8.224)**  4. pre-S1 del (3.615) | 1. wild-type (69.571)  2. pre-S2 del (nt 1-54) (18.482)  3. pre-S1+pre-S2 del (nt 2855-2872, 1-54) (7.712)  4. pre-S1 del (nt 2855-2872) (2.674) |
| 21 | 1. **pre-S2 del (41.477)**  2. **pre-S1+pre-S2 del (39.126)**  3. **wild-type (12.348)**  4. **pre-S1 del (7.048)** | 1. pre-S2 del (nt 1-54) (24.620)  2. pre-S1+pre-S2 del (nt 2855-2872, 1-54) (26.451)  3. wild-type (12.348)  4. pre-S1 del (nt 2855-2872) (4.978) |
| 22 | 1. **pre-S2 del (42.909)**  2. **pre-S1+pre-S2 del (27.915)**  3. **wild-type (17.564)**  4. **pre-S1 del (11.612)** | 1. pre-S2 del (nt 1-54) (38.091)  2. pre-S1+pre-S2 del (2855-2872, 1-54) (25.090)  3. wild-type (17.564)  4. pre-S1 del (nt 2855-2872) (8.072) |
| 23 | 1. **wild-type (52.661)**  2. **pre-S2 del (25.093)**  3. **pre-S1+pre-S2 del (11.424)**  4. **pre-S1 del (10.821)** | 1. wild-type (52.661)  2. pre-S2 del (nt 1-54) (18.682)  3. pre-S1+pre-S2 del (nt 2855-2872, 1-54) (8.344)  4. pre-S1 del (nt 2855-2872) (2.817) |
| 24 | 1. **wild-type (96.073)**  2. pre-S1 del (1.895)  3. pre-S1+pre-S2 del (1.202)  4. pre-S2 del (0.830) | 1. wild-type (96.073)  2. pre-S1 del (nt 2954-3097) (0.632)  3. pre-S1+pre-S2 del (nt 2855-2872, 1-54) (0.452)  4. pre-S2 del (nt 1-54) (0.382) |

**S1 Table. List of the pre-S genotyping results by TA cloning- and NGS-based analyses in 75 HBV-related HCC patients (continued)**

| Patient No. | NGS Result (Pre-S Del Type (%))^a^ | NGS Result (Pre-S Del Region (%))^b^ |
| --- | --- | --- |
| 25 | 1. **wild-type (93.547)**  2. pre-S1 del (4.345)  3. pre-S2 del (1.844)  4. pre-S1+pre-S2 del (0.265) | 1. wild-type (93.547)  2. pre-S1 del (nt 3026-3205) (0.869)  3. pre-S2 del (nt 1-54) (1.107)  4. pre-S1+pre-S2 del (nt 2855-2872, 2897-2923, 1-54) (0.180) |
| 26 | 1. **wild-type (97.921)**  2. pre-S1 del (1.388)  3. pre-S2 del (0.691)  4. pre-S1+pre-S2 del (0.000) | 1. wild-type (97.921)  2. pre-S1 del (nt 3067-3162) (0.040)  3. pre-S2 del (nt 3211-3216) (0.046)  4. pre-S1+pre-S2 del (0.000) |
| 27 | 1. **wild-type (60.195)**  2. **pre-S2 del (22.882)**  3. **pre-S1+pre-S2 del (9.280)**  4. **pre-S1 del (7.643)** | 1. wild-type (60.195)  2. pre-S2 del (nt 1-57) (22.473)  3. pre-S1+pre-S2 del (nt 2855-2872, 1-54) (4.815)  4. pre-S1 del (nt 3025-3126) (4.396) |
| 28 | 1. **pre-S1 del (75.113)**  2. **wild-type (11.506)**  3. **pre-S1+pre-S2 del (10.472)**  4. pre-S2 del (2.909) | 1. pre-S1 del (nt 2855-2872) (5.979)  2. wild-type (11.506)  3. pre-S1+pre-S2 del (nt 2855-2872, 1-54) (8.016)  4. pre-S2 del (nt 1-54) (2.747) |
| 29 | 1. **wild-type (97.730)**  2. pre-S1 del (1.691)  3. pre-S2 del (0.575)  4. pre-S1+pre-S2 del (0.004) | 1. wild-type (97.730)  2. pre-S1 del (nt 2855-2970) (0.217)  3. pre-S2 del (nt 1-12) (0.323)  4. pre-S1+pre-S2 del (nt 2855-2970, 1-54) (0.004) |
| 30 | 1. **wild-type (87.018)**  2. **pre-S1 del (11.703)**  3. pre-S2 del (1.113)  4. pre-S1+pre-S2 del (0.166) | 1. wild-type (87.018)  2. pre-S1 del (nt 3010-3075) (5.189)  3. pre-S2 del (nt 1-9) (0.392)  4. pre-S1+pre-S2 del (nt 3010-3075, 1-9) (0.046) |

**S1 Table. List of the pre-S genotyping results by TA cloning- and NGS-based analyses in 75 HBV-related HCC patients (continued)**

| Patient No. | NGS Result (Pre-S Del Type (%))^a^ | NGS Result (Pre-S Del Region (%))^b^ |
| --- | --- | --- |
| 31 | 1. **wild-type (78.709)**  2. **pre-S1 del (20.616)**  3. pre-S2 del (0.646)  4. pre-S1+pre-S2 del (0.029) | 1. wild-type (78.709)  2. pre-S1 del (nt 2910-3089) (9.181)  3. pre-S2 del (nt 1-15) (0.345)  4. pre-S1+pre-S2 del (nt 2910-3055, 3067-3089, 4-12) (0.007) |
| 32 | 1. **wild-type (98.105)**  2. pre-S1 del (1.152)  3. pre-S2 del (0.733)  4. pre-S1+pre-S2 del (0.010) | 1. wild-type (98.105)  2. pre-S1 del (nt 2855-2970) (0.060)  3. pre-S2 del (nt 1-9) (0.408)  4. pre-S1+pre-S2 del (nt 2855-2872, 1-54) (0.003) |
| 33 | 1. **wild-type (80.067)**  2. **pre-S2 del (9.477)**  3. **pre-S1 del (8.993)**  4. pre-S1+pre-S2 del (1.463) | 1. wild-type (80.067)  2. pre-S2 del (nt 1-12) (8.957)  3. pre-S1 del (nt 2866-3075) (7.322)  4. pre-S1+pre-S2 del (nt 2866-3075, 1-9) (0.871) |
| 34 | 1. **wild-type (80.175)**  2. **pre-S2 del (13.639)**  3. **pre-S1 del (5.169)**  4. pre-S1+pre-S2 del (1.017) | 1. wild-type (80.175)  2. pre-S2 del (nt 1-30) (4.394)  3. pre-S1 del (nt 2865-2975) (2.356)  4. pre-S1+pre-S2 del (nt 2866-2975, 1-29) (0.212) |
| 35 | 1. **wild-type (92.156)**  2. **pre-S1 del (7.022)**  3. pre-S2 del (0.793)  4. pre-S1+pre-S2 del (0.029) | 1. wild-type (92.156)  2. pre-S1 del (nt 2854-3018) (1.346)  3. pre-S2 del (nt 1-9) (0.323)  4. pre-S1+pre-S2 del (nt 2855-2872, 1-9) (0.004) |
| 36 | 1. **pre-S1+pre-S2 del (40.433)**  2. **pre-S1 del (34.174)**  3. **wild-type (20.822)**  4. pre-S2 del (4.571) | 1. pre-S1+pre-S2 del (nt 2855-2872, 1-54) (22.713)  2. pre-S1 del (nt 2854-2970) (22.956)  3. wild-type (20.822)  4. pre-S2 del (nt 1-54) (4.380) |

**S1 Table. List of the pre-S genotyping results by TA cloning- and NGS-based analyses in 75 HBV-related HCC patients (continued)**

| Patient No. | NGS Result (Pre-S Del Type (%))^a^ | NGS Result (Pre-S Del Region (%))^b^ |
| --- | --- | --- |
| 37 | 1. **wild-type (80.758)**  2. **pre-S2 del (12.910)**  3. **pre-S1 del (5.629)**  4. pre-S1+pre-S2 del (0.704) | 1. wild-type (80.758)  2. pre-S2 del (nt 1-54) (6.944)  3. pre-S1 del (nt 2855-2875) (4.209)  4. pre-S1+pre-S2 del (nt 2855-2875, 1-54) (0.253) |
| 38 | 1. **wild-type (61.320)**  2. **pre-S1 del (34.045)**  3. pre-S2 del (3.491)  4. pre-S1+pre-S2 del (1.144) | 1. wild-type (61.320)  2. pre-S1 del (nt 3021-3203) (23.818)  3. pre-S2 del (nt 6-41) (1.385)  4. pre-S1+pre-S2 del (nt 2855-2872, 2919-3126, 6-41) (0.163) |
| 39 | 1. **wild-type (97.172)**  2. pre-S1 del (1.957)  3. pre-S2 del (0.850)  4. pre-S1+pre-S2 del (0.021) | 1. wild-type (97.172)  2. pre-S1 del (nt 3021-3203) (0.353)  3. pre-S2 del (nt 1-12) (0.488)  4. pre-S1+pre-S2 del (nt 3078-3094, 6-41) (0.003) |
| 40 | 1. **wild-type (97.501)**  2. pre-S1 del (1.586)  3. pre-S2 del (0.908)  4. pre-S1+pre-S2 del (0.005) | 1. wild-type (97.501)  2. pre-S1 del (nt 3107-3202) (0.081)  3. pre-S2 del (nt 1-12) (0.529)  4. pre-S1+pre-S2 del (nt 3133-3136, 1-10) (0.005) |
| 41 | 1. **pre-S1 del (84.262)**  2. **wild-type (14.798)**  3. pre-S1+pre-S2 del (0.779)  4. pre-S2 del (0.161) | 1. pre-S1 del (nt 2858-2986) (82.700)  2. wild-type (14.798)  3. pre-S1+pre-S2 del (nt 2858-2981, 1-10) (0.268)  4. pre-S2 del (nt 1-33) (0.053) |
| 42 | 1. **wild-type (97.757)**  2. pre-S1 del (1.305)  3. pre-S2 del (0.923)  4. pre-S1+pre-S2 del (0.015) | 1. wild-type (97.757)  2. pre-S1 del (nt 3106-3207) (0.060)  3. pre-S2 del (nt 3211-3213) (0.124)  4. pre-S1+pre-S2 del (nt 3069-3118, 3211-3212) (0.004) |

**S1 Table. List of the pre-S genotyping results by TA cloning- and NGS-based analyses in 75 HBV-related HCC patients (continued)**

| Patient No. | NGS Result (Pre-S Del Type (%))^a^ | NGS Result (Pre-S Del Region (%))^b^ |
| --- | --- | --- |
| 43 | 1. **pre-S2 del (49.695)**  2. **wild-type (49.248)**  3. pre-S1+pre-S2 del (0.814)  4. pre-S1 del (0.242) | 1. pre-S2 del (nt 37-54) (43.929)  2. wild-type (49.248)  3. pre-S1+pre-S2 del (nt 3139-3142, 37-54) (0.049)  4. pre-S1 del (nt 3103-3200) (0.019) |
| 44 | 1. **wild-type (98.244)**  2. pre-S1 del (1.113)  3. pre-S2 del (0.643)  4. pre-S1+pre-S2 del (0.000) | 1. wild-type (98.244)  2. pre-S1 del (nt 3089-3202) (0.049)  3. pre-S2 del (nt 1-15) (0.319)  4. pre-S1+pre-S2 del (0.000) |
| 45 | 1. **wild-type (97.534)**  2. pre-S1 del (1.376)  3. pre-S2 del (1.076)  4. pre-S1+pre-S2 del (0.014) | 1. wild-type (97.534)  2. pre-S1 del (nt 3106-3207) (0.063)  3. pre-S2 del (nt 1-9) (0.769)  4. pre-S1+pre-S2 del (nt 3095-3150, 1-12) (0.007) |
| 46 | 1. **wild-type (97.735)**  2. pre-S1 del (1.534)  3. pre-S2 del (0.731)  4. pre-S1+pre-S2 del (0.000) | 1. wild-type (97.735)  2. pre-S1 del (nt 3138-3197) (0.111)  3. pre-S2 del (nt 1-9) (0.371)  4. pre-S1+pre-S2 del (0.000) |
| 47 | 1. **wild-type (78.334)**  2. **pre-S1 del (20.695)**  3. pre-S2 del (0.866)  4. pre-S1+pre-S2 del (0.105) | 1. wild-type (78.334)  2. pre-S1 del (nt 2895-3140) (15.887)  3. pre-S2 del (nt 1-9) (0.279)  4. pre-S1+pre-S2 del (nt 2895-3141, 1-19, 23-27) (0.023) |
| 48 | 1. **pre-S1 del (57.159)**  2. **wild-type (20.456)**  3. **pre-S2 del (13.666)**  4. **pre-S1+pre-S2 del (8.719)** | 1. pre-S1 del (nt 2968-3093) (47.067)  2. wild-type (20.456)  3. pre-S2 del (nt 1-54) (8.120)  4. pre-S1+pre-S2 del (nt 2968-3093, 1-54) (3.469) |

**S1 Table. List of the pre-S genotyping results by TA cloning- and NGS-based analyses in 75 HBV-related HCC patients (continued)**

| Patient No. | NGS Result (Pre-S Del Type (%))^a^ | NGS Result (Pre-S Del Region (%))^b^ |
| --- | --- | --- |
| 49 | 1. **wild-type (75.021)**  2. **pre-S2 del (21.983)**  3. pre-S1 del (2.327)  4. pre-S1+pre-S2 del (0.669) | 1. wild-type (75.021)  2. pre-S2 del (nt 1-1) (19.454)  3. pre-S1 del (nt 2855-2872) (0.704)  4. pre-S1+pre-S2 del (nt 2855-2872, 1-54) (0.606) |
| 50 | 1. **wild-type (98.169)**  2. pre-S1 del (1.138)  3. pre-S2 del (0.693)  4. pre-S1+pre-S2 del (0.000) | 1. wild-type (98.169)  2. pre-S1 del (nt 3138-3197) (0.067)  3. pre-S2 del (nt 1-9) (0.377)  4. pre-S1+pre-S2 del (0.000) |
| 51 | 1. **wild-type (97.734)**  2. pre-S1 del (1.546)  3. pre-S2 del (0.719)  4. pre-S1+pre-S2 del (0.000) | 1. wild-type (97.734)  2. pre-S1 del (nt 3104-3202) (0.165)  3. pre-S2 del (nt 1-9) (0.377)  4. pre-S1+pre-S2 del (0.000) |
| 52 | 1. **wild-type (98.167)**  2. pre-S1 del (1.192)  3. pre-S2 del (0.617)  4. pre-S1+pre-S2 del (0.024) | 1. wild-type (98.167)  2. pre-S1 del (nt 2854-3128) (0.132)  3. pre-S2 del (nt 1-9) (0.246)  4. pre-S1+pre-S2 del (nt 3022-3125, 1-55) (0.018) |
| 53 | 1. **wild-type (97.002)**  2. pre-S1 del (2.144)  3. pre-S2 del (0.849)  4. pre-S1+pre-S2 del (0.005) | 1. wild-type (97.002)  2. pre-S1 del (nt 3026-3205) (0.927)  3. pre-S2 del (nt 1-9) (0.528)  4. pre-S1+pre-S2 del (nt 3020-3119, 1-55) (0.005) |
| 54 | 1. **wild-type (96.687)**  2. pre-S1 del (2.625)  3. pre-S2 del (0.676)  4. pre-S1+pre-S2 del (0.013) | 1. wild-type (96.687)  2. pre-S1 del (nt 2855-2872) (1.117)  3. pre-S2 del (nt 1-12) (0.411)  4. pre-S1+pre-S2 del (nt 2855-2872, 1-17) (0.004) |

**S1 Table. List of the pre-S genotyping results by TA cloning- and NGS-based analyses in 75 HBV-related HCC patients (continued)**

| Patient No. | NGS Result (Pre-S Del Type (%))^a^ | NGS Result (Pre-S Del Region (%))^b^ |
| --- | --- | --- |
| 55 | 1. **wild-type (64.666)**  2. **pre-S1 del (34.582)**  3. pre-S2 del (0.493)  4. pre-S1+pre-S2 del (0.259) | 1. wild-type (64.666)  2. pre-S1 del (nt 2858-2986) (33.466)  3. pre-S2 del (nt 1-9) (0.303)  4. pre-S1+pre-S2 del (nt 2858-2981, 1-10) (0.069) |
| 56 | 1. **pre-S1 del (69.372)**  2. **wild-type (30.404)**  3. pre-S2 del (0.218)  4. pre-S1+pre-S2 del (0.005) | 1. pre-S1 del (nt 2856-3101) (66.854)  2. wild-type (30.404)  3. pre-S2 del (nt 1-12) (0.130)  4. pre-S1+pre-S2 del (nt 2984-3098, 1-10) (0.002) |
| 57 | 1. **wild-type (94.005)**  2. pre-S1 del (3.729)  3. pre-S2 del (1.824)  4. pre-S1+pre-S2 del (0.442) | 1. wild-type (94.005)  2. pre-S1 del (nt 2895-3188) (0.677)  3. pre-S2 del (nt 1-54) (1.029)  4. pre-S1+pre-S2 del (nt 2855-2872, 1-54) (0.253) |
| 58 | 1. **wild-type (95.575)**  2. pre-S1 del (2.481)  3. pre-S2 del (1.931)  4. pre-S1+pre-S2 del (0.013) | 1. wild-type (95.575)  2. pre-S1 del (nt 3039-3092) (0.590)  3. pre-S2 del (nt 1-12) (0.510)  4. pre-S1+pre-S2 del (nt 2854-2940, 1-144) (0.007) |
| 59 | 1. **pre-S2 del (64.182)**  2. **wild-type (32.367)**  3. pre-S1+pre-S2 del (2.975)  4. pre-S1 del (0.476) | 1. pre-S2 del (nt 15-56) (33.613)  2. wild-type (32.367)  3. pre-S1+pre-S2 del (nt 3088-3126, 15-56) (0.176)  4. pre-S1 del (nt 2923-3090) (0.073) |
| 60 | 1. **pre-S1 del (38.192)**  2. **wild-type (34.644)**  3. **pre-S2 del (19.195)**  4. **pre-S1+pre-S2 del (7.968)** | 1. pre-S1 del (nt 2854-2970) (27.998)  2. wild-type (30.404)  3. pre-S2 del (nt 1-54) (18.373)  4. pre-S1+pre-S2 del (nt 3025-3126, 1-57) (2.383) |

**S1 Table. List of the pre-S genotyping results by TA cloning- and NGS-based analyses in 75 HBV-related HCC patients (continued)**

| Patient No. | NGS Result (Pre-S Del Type (%))^a^ | NGS Result (Pre-S Del Region (%))^b^ |
| --- | --- | --- |
| 61 | 1. **pre-S1 del (40.086)**  2. **wild-type (34.919)**  3. **pre-S2 del (16.065)**  4. **pre-S1+pre-S2 del (8.930)** | 1. pre-S1 del (nt 2855-2965) (24.125)  2. wild-type (34.919)  3. pre-S2 del (nt 1-54) (15.565)  4. pre-S1+pre-S2 del (nt 3022-3126, 1-60) (2.911) |
| 62 | 1. **pre-S1 del (29.181)**  2. **wild-type (23.836)**  3. **pre-S2 del (23.645)**  4. **pre-S1+pre-S2 del (23.338)** | 1. pre-S1 del (nt 2856-2969) (17.015)  2. wild-type (23.836)  3. pre-S2 del (nt 1-54) (23.402)  4. pre-S1+pre-S2 del (nt 2855-2872, 1-54) (15.956) |
| 63 | 1. **pre-S1 del (52.401)**  2. **wild-type (42.979)**  3. pre-S2 del (2.312)  4. pre-S1+pre-S2 del (2.308) | 1. pre-S1 del (nt 2854-2970) (43.037)  2. wild-type (42.979)  3. pre-S2 del (nt 1-54) (1.977)  4. pre-S1+pre-S2 del (nt 2860-2880, 2954-3097, 1-15) (1.445) |
| 64 | 1. **pre-S1 del (41.155)**  2. **wild-type (37.573)**  3. **pre-S2 del (18.905)**  4. pre-S1+pre-S2 del (2.367) | 1. pre-S1 del (nt 2854-2970) (35.773)  2. wild-type (37.573)  3. pre-S2 del (nt 1-54) (18.494)  4. pre-S1+pre-S2 del (nt 2855-2970, 1-54) (1.676) |
| 65 | 1. **pre-S1 del (63.334)**  2. **wild-type (32.806)**  3. pre-S2 del (1.965)  4. pre-S1+pre-S2 del (1.895) | 1. pre-S1 del (nt 2854-2970) (33.450)  2. wild-type (32.806)  3. pre-S2 del (nt 1-54) (1.708)  4. pre-S1+pre-S2 del (nt 2855-2970, 1-54) (0.994) |
| 66 | 1. **wild-type (30.973)**  2. **pre-S1+pre-S2 del (27.774)**  3. **pre-S1 del (27.161)**  4. **pre-S2 del (14.091)** | 1. wild-type (30.973)  2. pre-S1+pre-S2 del (nt 2855-2872, 1-54) (24.398)  3. pre-S1 del (nt 2854-2970) (14.813)  4. pre-S2 del (nt 1-54) (13.846) |

**S1 Table. List of the pre-S genotyping results by TA cloning- and NGS-based analyses in 75 HBV-related HCC patients (continued)**

| Patient No. | NGS Result (Pre-S Del Type (%))^a^ | NGS Result (Pre-S Del Region (%))^b^ |
| --- | --- | --- |
| 67 | 1. **wild-type (46.914)**  2. **pre-S1 del (45.517)**  3. **pre-S2 del (6.834)**  4. pre-S1+pre-S2 del (0.735) | 1. wild-type (46.914)  2. pre-S1 del (nt 2854-2970) (37.666)  3. pre-S2 del (nt 1-54) (5.216)  4. pre-S1+pre-S2 del (nt 2854-2970, 1-54) (0.307) |
| 68 | 1. **pre-S1 del (43.130)**  2. **wild-type (41.965)**  3. **pre-S2 del (9.508)**  4. **pre-S1+pre-S2 del (5.397)** | 1. pre-S1 del (nt 2854-2970) (35.888)  2. wild-type (41.965)  3. pre-S2 del (nt 1-54) (9.239)  4. pre-S1+pre-S2 del (nt 2855-2872, 1-54) (4.160) |
| 69 | 1. **wild-type (36.868)**  2. **pre-S1 del (35.238)**  3. **pre-S2 del (21.130)**  4. **pre-S1+pre-S2 del (6.763)** | 1. wild-type (36.868)  2. pre-S1 del (nt 2854-2970) (29.499)  3. pre-S2 del (nt 1-54) (20.218)  4. pre-S1+pre-S2 del (nt 2855-2872, 1-54) (3.332) |
| 70 | 1. **wild-type (94.788)**  2. **pre-S1 del (4.643)**  3. pre-S2 del (0.455)  4. pre-S1+pre-S2 del (0.114) | 1. wild-type (94.788)  2. pre-S1 del (nt 2854-3021) (3.404)  3. pre-S2 del (nt 1-15) (0.192)  4. pre-S1+pre-S2 del (nt 2855-2872, 1-54) (0.086) |
| 71 | 1. **pre-S2 del (54.981)**  2. **wild-type (42.238)**  3. pre-S1+pre-S2 del (2.274)  4. pre-S1 del (0.507) | 1. pre-S2 del (nt 48-56) (52.982)  2. wild-type (42.238)  3. pre-S1+pre-S2 del (nt 3026-3205, 48-56) (0.916)  4. pre-S1 del (nt 2854-3021) (0.334) |
| 72 | 1. **wild-type (91.511)**  2. **pre-S1 del (7.310)**  3. pre-S2 del (0.807)  4. pre-S1+pre-S2 del (0.373) | 1. wild-type (91.511)  2. pre-S1 del (nt 2854-3021) (5.744)  3. pre-S2 del (nt 48-56) (0.294)  4. pre-S1+pre-S2 del (nt 2855-2872, 1-54) (0.322) |

**S1 Table. List of the pre-S genotyping results by TA cloning- and NGS-based analyses in 75 HBV-related HCC patients (continued)**

| Patient No. | NGS Result (Pre-S Del Type (%))^a^ | NGS Result (Pre-S Del Region (%))^b^ |
| --- | --- | --- |
| 73 | 1. **wild-type (52.297)**  2. **pre-S1+pre-S2 del (31.339)**  3. **pre-S1 del (12.863)**  4. pre-S2 del (3.502) | 1. wild-type (52.297)  2. pre-S1+pre-S2 del (nt 2855-2872, 3012-3086, 1-51) (8.935)  3. pre-S1 del (nt 2855-2872, 3012-3092) (6.480)  4. pre-S2 del (nt 1-51) (2.094) |
| 74 | 1. **wild-type (58.984)**  2. **pre-S2 del (34.533)**  3. pre-S1+pre-S2 del (4.497)  4. pre-S1 del (1.986) | 1. wild-type (58.984)  2. pre-S2 del (nt 1-57) (20.926)  3. pre-S1+pre-S2 del (nt 3026-3205, 2-55) (0.590)  4. pre-S1 del (nt 2944-3120) (1.128) |
| 75 | 1. **wild-type (97.112)**  2. pre-S1 del (2.178)  3. pre-S2 del (0.679)  4. pre-S1+pre-S2 del (0.031) | 1. wild-type (97.112)  2. pre-S1 del (nt 2854-3021) (0.641)  3. pre-S2 del (nt 25-54) (0.355)  4. pre-S1+pre-S2 del (nt 2855-2970, 25-54) (0.007) |

^a^The total frequency of pre-S gene DNA in each type of pre-S deletion was shown in descending order.

^b^The pre-S gene DNA with the highest frequency in each type of pre-S deletion was shown.

^c^The pre-S deletion type above the cut-off percentage (4.643) was shown in bold.

Abbreviations: del, deletion; nt, nucleotide.
