# Supplementary material for: Hepatitis B virus pre-S2 deletion (nucleotide 1 to 54) in plasma predicts recurrence of hepatocellular carcinoma after curative surgical resection
Source: PLoS One. 2020 Nov 25;15(11):e0242748. doi: 10.1371/journal.pone.0242748 (PMC7688176; doi:10.1371/journal.pone.0242748)
Supplement: S2 Table — (DOCX) [file pone.0242748.s002.docx]

**S2 Table. Clinicopathological correlation of pre-S deletion regions with survival after surgery in 75 HBV-related HCC patients**

| Characteristics^a^ | Alive (No. of Patients (%)) | Dead (No. of Patients (%)) | P value^b^ |
| --- | --- | --- | --- |
| Pre-S1 del (nt 2854-2970)  yes  no | 16 (100)  1 (6)  15 (94) | 59 (100)  9 (15)  50 (85) | 0.2425 |
| Pre-S1 del (nt 2855-2872)  yes  no | 16 (100)  2 (12)  14 (88) | 59 (100)  3 (5)  56 (95) | 0.2260 |
| Pre-S2 del (nt 1-54)  yes  no | 16 (100)  4 (25)  12 (75) | 59 (100)  13 (22)  46 (78) | 0.2482 |
| Pre-S1+pre-S2 del (nt 2855-2872, 1-54)  yes  no | 16 (100)  3 (19)  13 (81) | 59 (100)  9 (15)  50 (85) | 0.2694 |

^a^Only patients with available data were analyzed.

^b^P value was determined by the chi-square test.

Abbreviations: HCC, hepatocellular carcinoma; HBV, hepatitis B virus; del, deletion; nt, nucleotide.
