# Supplementary material for: Hepatitis B virus pre-S2 deletion (nucleotide 1 to 54) in plasma predicts recurrence of hepatocellular carcinoma after curative surgical resection
Source: PLoS One. 2020 Nov 25;15(11):e0242748. doi: 10.1371/journal.pone.0242748 (PMC7688176; doi:10.1371/journal.pone.0242748)
Supplement: S3 Table — (DOCX) [file pone.0242748.s003.docx]

**S3 Table. Univariate and multivariate analyses of pre-S deletion regions for overall survival in 75 HBV-related HCC patients**

| **Characteristics** | **Univariate Analysis** | | | **Multivariate Analysis** | | |
| --- | --- | --- | --- | --- | --- | --- |
|  | **HR** | **95% CI** | **P value** | **HR** | **95% CI** | **P value** |
| Age (years) (>50 vs. ≤50) | 0.764 | 0.270-2.159 | 0.6109 |  |  |  |
| Gender (men vs. women) | 1.462 | 0.191-11.179 | 0.7143 |  |  |  |
| Smoking (yes vs. no) | 1.004 | 0.357-2.821 | 0.9947 |  |  |  |
| Alcohol (yes vs. no) | 2.048 | 0.760-5.524 | 0.1566 |  |  |  |
| HBsAg (positive vs. negative)^a^ |  |  |  |  |  |  |
| HBeAg (positive vs. negative)^b^ | 0.635 | 0.083-4.861 | 0.6616 |  |  |  |
| HBV genotype (B vs. C) | 1.296 | 0.292-5.752 | 0.7332 |  |  |  |
| HBV DNA (copies/mL) (>1×10^4^ vs. ≤1×10^4^)^c^ | 1.682 | 0.561-5.043 | 0.3537 |  |  |  |
| Albumin (g/dL) (>3.8 vs. ≤3.8) | 0.425 | 0.135-1.341 | 0.1444 |  |  |  |
| AST (U/L) (>34 vs. ≤34) | 1.323 | 0.298-5.864 | 0.7126 |  |  |  |
| ALT (U/L) (>40 vs. ≤40) | 0.849 | 0.307-2.349 | 0.7530 |  |  |  |
| AFP (ng/mL) (>400 vs. ≤400) | 2.542 | 0.937-6.895 | 0.0669 |  |  |  |
| Tumor size (cm) (>5 vs. ≤5) | 1.605 | 0.581-4.437 | 0.3615 |  |  |  |
| Tumor encapsulation (yes vs. no)^d^ | 0.386 | 0.124-1.199 | 0.0997 |  |  |  |
| Lymph node involvement (yes vs. no) | 0.513 | 0.067-3.899 | 0.5186 |  |  |  |
| Portal vein thrombosis (yes vs. no) | 1.981 | 0.444-8.834 | 0.3701 |  |  |  |
| Vascular invasion (yes vs. no) | 1.930 | 0.680-5.480 | 0.2169 |  |  |  |
| Distant metastasis (yes vs. no) | 3.336 | 0.924-12.040 | 0.0658 |  |  |  |
| Steatosis grade (2/3 vs. 0/1)^e^ | 21.354 | 1.329-343.018 | 0.0307* |  |  |  |
| Metavir inflammation score (2/3 vs. 0/1)^f^ | 1.474 | 0.284-7.645 | 0.6438 |  |  |  |
| Ishak fibrosis score (4/5/6 vs. 0/1/2/3)^g^ | 1.153 | 0.324-4.100 | 0.8263 |  |  |  |
| Child-Pugh cirrhosis score (B/C vs. A) | 2.953 | 1.044-8.356 | 0.0413* | 2.974 | 1.035-8.549 | 0.0430* |
| CLIP score (4/5/6 vs. 0/1/2/3) | 5.233 | 0.675-40.579 | 0.1133 |  |  |  |
| BCLC stage (C/D vs. A/B) | 1.314 | 0.296-5.834 | 0.7199 |  |  |  |
| AJCC TNM stage (IIIA/IIIB/IIIC/IVA/IVB vs. I/II) | 3.113 | 1.053-9.201 | 0.0400* | 3.146 | 1.041-9.510 | 0.0423* |
| Antiviral therapy after surgery (yes vs. no) | 0.746 | 0.279-1.993 | 0.5590 |  |  |  |
| Pre-S1 del (nt 2854-2970) (yes vs. no) | 0.659 | 0.087-5.015 | 0.6870 |  |  |  |
| Pre-S1 del (nt 2855-2872) (yes vs. no) | 2.954 | 0.645-13.518 | 0.1628 |  |  |  |
| Pre-S2 del (nt 1-54) (yes vs. no) | 1.669 | 0.519-5.369 | 0.3903 |  |  |  |
| Pre-S1+pre-S2 del (nt 2855-2872, 1-54) (yes vs. no) | 1.663 | 0.467-5.920 | 0.4322 |  |  |  |

^a^There were no patients negative for HBsAg for analysis.

^b^Only 71 patients with available data were analyzed.

^c^Only 74 patients with available data were analyzed.

^d^Only 62 patients with available data were analyzed.

^e^Only 25 patients with available data were analyzed and thus excluded from multivariate analysis.

^f^Only 44 patients with available data were analyzed.

^g^Only 56 patients with available data were analyzed.

*, P value<0.05.

Abbreviations: HR, hazard ratio; CI, confidence interval; del, deletion; nt, nucleotide.
